# Spirit of the British and American Periodicals, &c.

**Published:** 1843-04-01

**Authors:** 


### Spirit of the British and American Periodicals.

### DR. GARDNER ON CALOMEL.

"IN Dr. Pereira's well-known work, the article *Chloride of Mercury*, sets forth very strikingly a great discrepancy existing in medical authors upon its therapeutic uses and effects. Its employment in hydrocephalus—Asiatic cholera—for the general purposes of a mercurial—and as a purgative, has been watched by innumerable practitioners, and their reports, compiled from very numerous sources, present assertions so directly opposed to each other, that Dr. Pereira says they appear to him to be irreconcilable. He quotes a passage from Gölis, to which I must invite your attention :—

"'Wytt, Colier, Quin, Wilmer, Liel, and others,' says Gölis, 'gave calomel internally in far larger doses, as two, three, and more grains at a time, and continued its use many days in the same dose, without considering the many evacuations from the alimentary canal, or the violent cholic pains; and they affirm, that they have never remarked from the effect of this agent given in these large doses, any bad consequences in the abdomen. Melancholy experience compels me to contradict them. Many times I saw, under those large and long-continued doses of calomel, the hydrocephalic symptoms suddenly vanish, and inflammation of the intestines arise, which terminated in death. Still oftener I observed this unfavourable accident from an incautious use of calomel in croup; viz. where all the frightful symptoms of this tracheal inflammation, which threatened suffocation suddenly vanish, and enteritis develop itself, which passed rapidly into gangrene, and destroyed the patient.'

"After this quotation Dr. Pereira proceeds to collect cases in which calomel has been found to act as an irritant poison, and others in which very large doses have produced effects which have led to its being denominated a *sedative*. This class of effects has been witnessed more especially in the treatment of cholera, and the authorities upon which the reports depend are so unquestionable, that it must be admitted as an established fact, that more cases of severe cholera have recovered under the use of prodigious doses of calomel than by any other means.

"'We have the concurrent testimony of many practitioners that, in yellow fever, cholera, and other dangerous diseases, calomel, in doses of a scruple and upwards, allays vomiting and purging, and is actually sedative.'

"'I do not pretend,' says Dr. Pereira, 'to reconcile these cases with those recorded by Kellewig, Vagritius, Ledelius, Hoffman, and Gölis; in fact, they appear to me irreconcilable. Dr. Christison, however, suggests that, in those cases in which violent effects occurred, the calomel might contain corrosive sublimate.'

"These discrepancies admit of another explanation, which will suggest, I hope, still further practical investigations than those I am about to relate.

"Many years ago it happened that my attention was powerfully drawn to the subject of hydrocephalus, and I was led to study carefully the earliest symptoms which indicate its approach. A well known circumstance in the history of this disease, namely, the great probability of its occurrence to other children in a family where one has been cut off by it, induces parents to watch very carefully for such symptoms as they are instructed to look for, and to seek our aid early. The effusion of fluid into the cavities or upon the surface of the brain, which gives a name to the disease, was long since admitted to be the result of a previous state of inflammation, and afterwards it was established that it is seldom that the symptoms of inflammation occur without a longer or shorter continuance of another state, to which the term *erythism* was applied by Dr. Whitlock Nicholl: more commonly, however, this state is designated *irritation*. An inseparable concomitant of that state of the infantile brain which is so called, is the occurrence of *white stools*, an appearance manifestly depending upon an altered condition of the bile. It is an interesting and important question, which of these circumstances precedes the other? Is the disturbance in the functions of the brain the *cause* or the effect of this remarkable action of the liver? We can prove that it is the brain, which, from whatever cause it may take on this diseased action, produces a secondary affection of the liver, discolouring its secretion; and the proof is this—that the brain is subject to a deviation from its normal state, antecedent to and irrespective of the irritation or erythism, an irregularity in its growth, traceable in the external form of the head to which, in a published pamphlet, I have applied the term *kephalosis* or morbid head. In children whose brain is developed in an irregular or abnormal manner, the state called erythism is peculiarly liable to occur, and its concomitant condition of the liver giving rise to white stools. Thus, it is certain the brain-affection is the *cause* not the *effect* of this symptom. It is also important to remark, that the white stools arising from brain-irritation are almost always associated with diarrhœa.

"Now, laying aside the authorities for the use of the chloride of mercury in hydrocephalus and inflammation, its old repute for an action directly upon the liver naturally gave rise to its application in this state of disease. It was, however, found by many practitioners to add to, rather than diminish the evil, by purging, irritating the abdominal viscera, and producing a disease worse than that it was intended to remove. In my first attempts to treat these cases with calomel, I met with such a decided check from these untoward symptoms, that for some time I laid aside its use altogether, and tried to accomplish the desired purpose by such general means as medical authors have recommended. Accident, however, led me to notice a very remarkable difference in the physical appearance of two specimens of calomel, and at the same time a remark occurred to me, made by Dr. Plummer, in his paper in the Edinburgh Medical Essays, where he recommends his well-known pill, 'that it is in vain to look for the beneficial effects of that pill, unless the materials are well levigated together and for a considerable time.' This suggested to me that the irritant effects of my calomel might have depended upon some difference in its physical state. I therefore resolved to try the new specimen, which was whiter in colour and considerably less in gravity; and in order to insure its minute subdivision, I had it triturated for a long time with an equal quantity of chalk. The result was, that a totally different effect followed its use in these cases of brain-irritation in children; instead of adding to the irritation and increasing the purging, the first effect of a large dose—two, three, or more grains upon an infant, was always what is called sedative; that is, whereas the morbid restlessness or sleeplessness which is the characteristic of the disease, might have lasted one, two, or more days and nights, a long quiet sleep followed immediately on its exhibition, and upon following it up with repeated doses, the stools became less frequent, and changed at first to a green colour, and afterwards became natural both in colour and texture, and the child was restored to health. It is upwards of ten years since I recognised this important difference in the therapeutic effects of different kinds of calomel, during which time I have administered it in hundreds of cases without a single instance of the occurrence of injurious effects. And it happens constantly that parents apply to me for these white powders, under the impression that they contain some sleeping drug, more safe than opium or any of the usual narcotics.

"I have been content to employ the calomel I thus found to answer my purpose without further enquiry, until, very recently some circumstances drawing my attention again to the subject, I referred to Dr. Pereira and other writers, and finding ths discrepancies beforementioned, I deemed the subject worthy of further research.

"In the first place, to determine the question as to the difference in the physical state of the various specimens, I examined several under the microscope, and found that crystalline fragments could be detected in some specimens in considerable proportions, and of far larger size than in others, every specimen I could procure varying much in this respect, although all of them were perfectly impalpable. The fine white light calomel I have been accustomed to employ, giving much fewer and far more minute crystalline particles than the calomel procured at Apothecaries' Hall, and of some celebrated makers. Being desirous of ascertaining by what process the former was prepared, I wrote to Mr. Davy, the maker, who kindly informed me—

"'That his method of making chloride of mercury consists in forming the sulphate in the usual manner, and after mixture with chloride of sodium, subjecting it to distillation into a dry chamber, repeating the process two, three, or more times, until the chloride comes over quite white, and entirely free from perchloride. The advantages of this process Mr. Davy considers to be, its avoiding the powdering and washing, and yet obtaining a perfectly pure chloride in the state of an impalpable powder of a very white colour.'"*

---

### REMARKS ON THE EFFICACY OF VACCINATION IN THE PARISH OF BIRMINGHAM. By HENRY KNIGHT, Esq.†

Alluding to the necessity which existed for the late Vaccination Act, Mr. Knight makes the following statements with regard to the amount of small-pox in Birmingham before and after the coming into effect of the Act.

During the last three months of 1840, small-pox prevailed to a frightful extent, amounting to full 11 per cent. of the entire number of deaths in the parish; and in the most central district, 23⅓ per cent. died of small-pox. From the 1st of January to the 7th of February, in 1841, nine deaths from small-pox were registered in the same district, being equal to 20 per cent. of the whole number of deaths.

At the commencement of the year 1841, the provisions of the Vaccination Act were carried out, and, apparently, with the most happy results. During 1841, 1481 persons were successfully vaccinated, and in 1842, the number was 850.

The consequence was, that during the whole of the year 1842, but 10 deaths were registered in the entire parish of Birmingham as caused by small-pox, being about 1 in every 358 deaths, instead of 1 in every 9 deaths, as was the case in the quarter ending Dec. 31, 1840. And further, not one of these 10 deaths has occurred since the month of April last, so that for many months it would appear that no death has been caused by small-pox in that parish.

---

### CONGESTIVE PNEUMONIA, CONSEQUENT UPON OPERATIONS, &c. By MR. ERICHSEN.‡

In a paper lately read to the Medico-Chirurgical Society, Mr. Erichsen contends, that in the diseases and injuries which come under the care of the surgeon, and

---

* Pharmaceutical Journal, Vol. 2, No. 9.
† Provincial Medical Journal, March 11th.
‡ Medical Times, Feb. 18.

in operations generally, a form of inflammation of the lungs, characterized by its asthenic nature, and different from that which is dependent on the absorption of pus, is a frequent attendant. This inflammation differs from active idiopathic pneumonia, and resembles more that condition of the lungs occasionally found in typhus, and other diseases of debility. Congestive pneumonia, however, is specially marked by an engorged and condensed condition of a considerable part, and that most frequently the inferior and posterior part of these organs. The blood, under the influence of depressing causes, such as confinement in the recumbent posture in impure atmosphere, and the irritative fever consequent upon wounds or profuse discharges, stagnates in the lungs; irritation is set up, passive inflammation is excited.

In the first stage, the affected parts are of a livid, violet or purple-mottled colour, heavy, compact, but friable, readily breaking down into a grumous pulp, and scarcely crepitating when pressed upon, but exuding a very considerable quantity of their spumous frothy fluid. In the second stage, the tissue is more dense, but still friable, it does not crepitate, but sinks in water, and when cut into, presents a smooth uniform black aspect, caused by a highly gorged state of the capillary net work of the lungs compressing the air-cells, which are either empty, or contain, almost, a thin serous fluid.

In order to establish this proposition, Mr. Erichsen presents a table containing a record of 62 post-mortem examinations of the lungs in patients who had been treated in the surgical wards of the University College Hospital. He arranges his cases into four classes, and finds, first, that of those in which the presence of a pneumonia was evinced by the diseased condition being confined to one lung, by its having advanced to solidification, or by its being combined with inflammation of the pleuræ or bronchial mucous membrane, there are 28 cases, or nearly one-half of the total number. Secondly, that of doubtful cases, in which the lungs presented the characters common to the first stage of pneumonia, and to passive congestion, without their being collateral signs to establish the diagnosis, there are 11. Thirdly, that of cases in which the lungs were diseased, but not inflamed or congested, there are 9. Lastly, that of cases in which the lungs were found to be perfectly healthy, there are 14. He further states that of the 28 cases included in the first class, the pneumonia had advanced in 17 to the second stage of hepatisation.

---

### A New Mode of Employing the Nitrate of Silver in certain Ophthalmic Affections. By M. Desmarres.*

M. Desmarres is of opinion that there is a similitude of action on our tissues between astringents and cold water, between caustics and intense cold. That consequently nitrate of silver acts as cold water if applied weak, as intense cold if applied strong. That if, therefore, nitrate of silver is applied in a weak form, much re-action is to be feared, giving rise to the necessity of having recourse to the antiphlogistic treatment. At the same time it is not generally expedient to employ it as a caustic, because, an eschar being once formed, the subjacent tissues are removed from the action of the medicine. The collyrium, therefore, which M. Desmarres employs is of the strength of from 7 to 15 grains of nitrate of silver to 2½ drachms of water, according to the amount and duration of the photophobia, this, which but seldom whitens the mucous membrane, is to be instilled into the eye every half hour during 24 hours without interruption. During the first two or three hours, considerable pain is experienced, this being

---

* Medical Gazette, March 10th.

the usual period necessary for the establishment of tolerance; after that time, however, the pain will subside. In this manner the diseased eye is not liable to re-action. At the end of six hours, M. Desmarres examines the eye to see whether re-action tends to supervene; if it does not, the collyrium is continued; if it does, the strength of the lotion is increased. In some cases, fomentations of iced water are recommended to assist the action of the nitrate of silver.

After 24 hours, re-action is no longer to be dreaded, but the intolerance of light is not always completely subdued, though the ingestion of the external tunics is usually diminished; in this case the strength of the collyrium ought to be increased, and after 48 hours the ophthalmia is at its second stage; the acute form no longer exists. The instillations are then repeated less often, at length discontinued, and replaced by appropriate general treatment.

By this method, long continued antiphlogistic treatment is avoided, which is of great consequence, especially in scrofulous persons; the increase of inflammation is arrested, and the intolerance of light quickly subdued. Moreover, in case of relapse, this treatment can always be again had recourse to, without inconvenience.

This remedy is not intended to supersede all others; when the photophobia is relieved, all is not accomplished; it is then that the experienced practitioner is called upon to employ remedies capable of restoring the organ to its normal condition.

---

### PORTUGUESE PRACTICE.*

CASE I.—*Ascites complicated with Anasarca and Tympanitis. By Signior J. M. Alvito.*

A woman, aged 41, labouring under these diseases, was cured in four months, chiefly by the endermic application of squill and digitalis, compresses to the abdomen, blisters to the thighs, dry frictions, rubbing-in of volatile camphor liniment, and ligature of the leg. In this case the dropsy was purely asthenic.

CASE II.—*Twin-birth, accompanied by Convulsions. By Signior D. B. S. Cadet.*

A young woman, aged 38, well formed, was seized with the pains of her first labour, accompanied by convulsions. On the following morning she was delivered of a living child, but without the placenta. In spite of repeated bleedings, &c. she remained in the same spasmodic state till the following morning, when she was delivered of a second child and also of the placenta. When seen by our author, she was still in an apoplectic state: the cervix uteri was but slightly open; the hæmorrhage was small. The prescriptions were, twenty-four leeches to the abdomen, copious venesection, sinapisms, &c. In the evening another blister was ordered. The patient improved, and on the fifth day the placenta came away. The reporter observes that he took the violent constriction of the os uteri to be the cause of the retention of the placenta, and employed antiphlogistics with the intention, if they did not succeed, of removing the after-birth, by artificial enlargement of the os uteri. [The translator doubts whether the recovery occurred by means of, or in spite of, the bleedings.]

CASE III.—*Serous Vesicular Swelling of the Liver. By Jos. Pereira e Sousa.*

A foot soldier, æt. 40, entered the hospital with what appeared to be ascites of long standing, combined with recent anasarca. Swelling and pain in the hepatic region, inducing the belief that there had been hepatitis originally, led to

---

* Medical Gazette, Jan. 6.

the employment of antiphlogistic remedies. At the end of some weeks the man died.

The section displayed an enormous cystic tumour, which hung down from the right anterior edge of the liver, and was covered by the peritoneum. It contained from 28 to 30 pounds of dark yellow serum, with many albuminous flakes. The liver was pale, not enlarged. It is said, in the observations which follow, that hepatic diseases are very common in Portugal, and that their study and treatment ought consequently to be much further advanced than they are.

The cystic tumour in this instance is remarkable for its enormous size.

---

### On the Nature and Cure of Blindness produced by Oil of Vitriol. By Robert D. Thomson, M.D.*

It is the general opinion at the present day, that the basis of animal matter is a substance named protein; this substance appears to be a base, and combines with acids. When sulphuric acid is brought into contact with the conjunctiva lining the cornea, which contains as its basis *protein,* sulphoproteic acid is formed, and opacity of the transparent cornea takes place. Our author found, by experiments on dead animals, that this layer of sulphoproteic acid may be removed from the cornea by means of a sharp-edged knife; that fresh application of sulphuric acid will produce fresh formation of sulphoproteic acid, which may be again scraped away, and so on until the whole of the cornea disappears. The question to be decided then was, whether this operation was practicable on the living body. The following experiment was made: a dog was caught, a glass rod dipped in vitriol was rubbed over the cornea, white opacity resulted in a few seconds. The action was allowed to continue for two minutes, the acid being prevented from spreading to the eyelids. The conjunctiva was then removed by means of a pair of scissors, assisted by a scalpel and forceps, and the denuded cornea was then scraped till it appeared to be deprived of its white opacity. Slight dulness remained; in a day or two the perfect transparency of the membrane was restored, and the animal lived for many weeks with complete vision of the eye.

The author thinks he has seen cases in which vision might have been restored, had this operation been performed immediately after the receipt of the injury.

---

### Fortitude of the Mind during Operations.†

With reference to the late well-known mesmeric amputation, Sir R. Dobson relates the following instances in which operations were borne without eliciting any exclamations of suffering.

When the late Sir Thomas Thompson lost his leg in action, it is well known that he was singing during the time the operation was being performed. In the burial ground at Greenwich Hospital is a monument to a seaman who was wounded at Trafalgar; the epitaph relates that, "while the amputation was performing, he was exultingly singing the patriotic song of Rule Britannia." Another seaman in Greenwich Hospital while losing his leg, said to the surgeon, "Avast a bit while I take a pinch of snuff," coolly took the box out of his pocket, and after having offered a pinch to the assistant-surgeon, took one himself, and the operation was finished without his having uttered a groan.

---

* Medical Gazette. Jan. 6th. † Lancet, January 21.

In none of these cases, it is almost needless to say, was any mesmeric influence employed.

---

### ACCIDENTS WITH HARD RINGS.*

On the subject of the removal from the fingers of case-hardened steel rings, such as are used for common silk purses, and which no file can touch, no nippers divide, Mr. Thomson suggests the employment of a simple instrument, called a clockmaker's hand-vice, the chops of which are narrow enough to go between any of the finger-joints. By means of a screw, the chops may be closed slowly and just enough to break the ring. This method is only applicable or in fact necessary when the ring is quite hard. If the steel is at all softened, it may be cut by a hard file.

---

### ON THE PRESERVATION OF INFANTS BY INOCULATION. TRANSLATED FROM THE CHINESE BY W. LOCKHART, M.D.†

It will probably be remembered that Dr. Lockhart published a short time ago a translation of a Chinese Treatise on Midwifery; the present sketch is from the pen of the same author. We shall proceed to give some short extracts from it.

Small-pox arises from poison introduced into the system from the mother's womb; but its form and character is determined by the external impression. "The truth is, that the breaking out of small-pox depends on external impressions; thus disease induces disease, and this eruption makes its appearance."

Inoculation does not appear to be of very recent invention; "it is handed down from the time of Chin Tzung, of the Sung dynasty (A. D. 1014), and was invented by a philosopher of Go-mei-Shan, in Sze-Chuen." The author is very indignant against those who are unwilling to be inoculated; it is, he says, "just as if a person, who had indigestion, were to refuse to eat food." The results of inoculation are stated to be very favourable; "out of ten thousand cases not one casualty will occur. Perhaps in one hundred thousand one or two fatal cases may accidentally be met with, arising perchance from bad management in the family, or because the inoculator has not carefully examined into the state of the patient."

*Modes of Inoculation.*—"Several pustules are to be chosen, and rubbed down in a cup with a piece of bamboo, or a twig of willow; then taking a fragment of cotton, rolled up into the shape of a date-seed, with this absorb the whole of the moistened lymph, and insert it into one of the nostrils, in the boy *on the left side*, in the girl *on the right*." "This is called the *watery inoculation;* it is safe, and may always be trusted." This pellet is to remain in the nostril for twelve hours, at the end of which time, "the spirit of the lymph enters the body, and gradually diffuses itself through the fine parenchymatous viscera." There are also other modes, the cotton may be moistened with variolous lymph; this is called the *lymph inoculation.* "The pustules being broken, you take the lymph; the child must be ordered to hold its mouth, but this is difficult to bear." Or you may put on the clothes of a child affected with this disease and soiled with lymph; this is called *clothes inoculation.* Or you may dry and pound the crusts, and then blow the powder up the nostrils; this is called *dry inoculation;* "and by following one or other of these plans the inoculation will surely take effect."

---

* Lancet, January 21. † Dublin Journal, March.

*Mode of Action.*—The variolous matter takes rather a curious course. When the lymph is placed in the nose, its influence is communicated to the lungs, the lungs govern the hair and skin; the lungs transfer the poison to the heart; the heart governs the pulse and transfers the poison to the spleen; the spleen governs the flesh and transfers it to the liver; the liver governs the tendons and transfers it to the kidneys; the kidneys govern the bones." "The poison of small-pox lies hid originally in the marrow of the bones;" but when it manifests itself, it spreads in the following order:—"the poison passes wholly from the marrow into the tendons, and the poison which was concealed in the kidney is dissipated; from the tendons it passes into the flesh, and the poison of the liver is dissipated; from the flesh it passes into the blood-vessels, and the poison of the spleen is dissipated; from the blood-vessels it passes into the skin and hair, and the poison of the heart is dissipated; from the skin it passes wholly into the pustules, and the poison of the lungs is dissipated." The variolous lymph thus traverses the whole of the viscera to arrive at the bones, where it dislodges the small-pox poison which had remained snugly concealed there from birth, and makes it follow the same course out of the body, which the lymph had taken in entering.

In inoculating, however, there is one most important circumstance which ought always to be attended to, namely, *the choice of lucky days.* "The eleventh day of the moon ought to be avoided, for at that time a person's spirit is in the pillar or septum of the nose; also the fifteenth day of the moon, because on that day the spirit is in the sides of the body."

---

### Relative Frequency of Affections of Different Organs in Cases of Burns.*

Mr. Erichsen has contributed to the Medical Gazette some valuable observations on the lesions of internal organs, consecutive to burns. The relative frequency with which different organs are affected at different ages has been set forth in a tabular form. Of the cases that he refers to, 29 occurred below the ages of 14 years, and 20 above that epoch.

Of the 29 cases, below the age of 14,—

| | | |
|---|---|---|
| The brain and it membranes were not examined in | 8 | |
| They were healthy in . . . . . . . . | 4 | |
| ———— diseased in . . . . . . . . | 17 | or 80·9 per cent. |
| The thoracic viscera were not examined in . . | 5 | |
| ———— healthy in . . . . . | 6 | |
| ———— diseased in . . . . . | 18 | or 78·2 per cent. |
| The abdominal viscera were not examined in . | 2 | |
| ———— healthy in . . . . . | 6 | |
| ———— diseased in . . . . . | 21 | or 77·7 per cent. |

Of the 20 cases that occur above the age of fourteen:—

| | | |
|---|---|---|
| The brain and its membranes were not examined in | 5 | |
| ———— healthy in . . . | 0 | |
| ———— diseased in . . . | 15 | or 100 per cent. |
| The thoracic viscera were not examined in . . | 3 | |
| ———— healthy in . . . . . | 3 | |
| ———— diseased in . . . . . | 14 | or 82·3 per cent. |
| The abdominal viscera were affected in . . . | 13 | or 81·2 per cent. |
| ———— healthy in . . . . | 3 | |
| ———— not examined in . . . | 4 | |

---

* Medical Gazette, Jan. 20, 1843.

### ON THE FRUITS OF HEMLOCK AND ANISE. By JONATHAN PEREIRA, M.D.*

The resemblance between the fruits (usually called seeds) of anise and hemlock, is such that the one may readily be mistaken for the other by a superficial observer. A case of poisoning which has recently occurred in France from an error of this kind, induced Dr. Pereira to lay the following account of the distinctive characters of the two fruits before the Pharmaceutical Society.

The following is a brief sketch of the case referred to.

A gentleman who had at various times derived benefit from the use of an infusion of anise, took, on one occasion, his usual medicine without deriving the accustomed relief. On the contrary, he experienced after its use various alarming symptoms, such as extreme uneasiness, followed by slowness of pulse, coldness of extremities, and other complaints, all of which ceased after copious vomiting. The physician who had been called in, having declared that the symptoms resembled those of poisoning, the remains of all the substances which had been used at table were examined, but without any noxious ingredient being detected. A fresh infusion of anise was prepared and taken, the same symptoms followed. The water, sugar, and anise were now carefully examined. In the two first nothing injurious was found, but in the anise some suspicious fruits were detected, which Professor Richard declared to be those of hemlock. They were recognized by the five crenulated ridges which each mericarp or half-fruit presented.

1. *Hemlock Fruits.*—The so-called hemlock seeds of the shops consist principally of half-fruits (*mericarps*), with some few entire fruits (*cremocarps*), rarely with a portion of the stalk attached. The entire fruits are roundish, ovate, compressed at the sides, about one line in length, and seldom exceeding one line in breath. The half-fruits are arched on the outer side. Each has five prominent equal ridges, the lateral ones being marginal. All the ridges have a notched appearance, that is, they are wavy, and have small convex teeth. Examined by the microscope, the elevations have a tuberculous appearance, sometimes being placed on the edge, at others somewhat on the side of the ridge. The channels or furrows are striated, but have no vittæ. The inner face of each mericarp presents a longitudinal line, or deep narrow furrow, indicative of the involute albumen. The seed in shape somewhat resembles the coffee-seed of the shops, being arched or convex on the outer side, and nearly flat with a longitudinal furrow on the inner side; but it is somewhat tapering superiorly. The albumen is rolled inwards, at the edge or side (*involute*), so as to form a longitudinal furrow. When examined by the microscope, it presents a granular appearance.

2. *Anise Fruits.*—These are called in the shops aniseed. They consist usually of entire fruits, each of which is generally provided with a stalk, of from two to four lines. Sometimes one-half of the fruit is abortive, or only imperfectly developed. The entire fruits are ovate, and slightly contracted at the sides: from one line to one and a half long, and about three-quarters of a line broad, and slightly pubescent. On splitting the entire fruit into its halves, we see the bifid, free carpophorus to which each is attached. Each half-fruit has five paler filiform, equal, entire ridges; the lateral ones being marginal. The channels are multivittate. The seed is gibbous, convex on its outer side, flattish on its inner face (commissure): its albumen is flat, being neither involute nor convolute.

---

* Pharmaceutical Journal, Nov. 1, 1842.

### On the Use of Nitric Acid in certain Hæmorrhoidal Affections. By John Houston, M.D.*

The form of hæmorrhoidal disease in which Dr. Houston chiefly recommends the use of nitric acid, as an escharotic, is that state of the mucous membrane to which the term " vascular tumor " is applied. Of this there are two varieties, which, although differing somewhat in origin and nature, admit of cure by the same means. One of these is that to which the term " erectile " has been given; the other—a congested, hypertrophied, and tender state of the membrane, the result of irritative or inflammatory action.

The first is regarded by many as a sort of aneurism by anastomosis of the small vessels of the mucous membrane and sub-mucous tissue exclusively, and may be independent from the first of varices, of the general veins about the anus.

The second variety is of a chronic inflammatory nature, and may be compared to the red bleeding surface seen on the mucous membrane of the eyelids in old cases of conjunctivitis. As in the foregoing variety, there is no relief for this affection but in the destruction of the morbid growth.

Dr. Houston is of opinion, therefore, that, the seat of the baneful part of the affection lying, as he concludes it does, on the surface, it is better to adopt such means as may remove that surface, *per se*, without extending beyond it, rather than by other, and more severe operations, run the risk of wounding large vessels, or of producing painful and dangerous symptoms.

These means are found in pure nitric acid, which removes, with little pain, and without danger, the tender, tumid, and bleeding surface; and, in the cicatrisation which rapidly follows, a radical cure is effected.

The application of the acid may be made in the following manner. Let the patient strain as at the night-chair, so as to bring the tumors fully into view; and, while they are so down, let him either lean over the back of a chair, or lie down, in the bent posture, on the side on which the disease exists, with the buttocks over the edge of the bed. Let a piece of wood cut into the shape of a dressing-case spatula, be dipped in the acid, and then, with as much of the acid adhering to it as it will carry, without dripping, let it be rubbed on the tumor to the extent desired. The due effect of the acid on the part is shewn by its changing it to a greyish-white colour. If a superficial slough be all that is required, a single application will suffice; if a more deep one, then two or three applications of the wood dipped in the acid may be made in quick succession; and immediately afterwards, the part must be well smeared over with olive oil, provided beforehand for the purpose. The prolapsed parts should then be pushed back within the sphincter, the patient put to bed, and an opiate administered. The pain of the application is sharp and burning at first, but soon goes off, and does not again return in the same form. A general uneasiness about the anus on motion, together with a slight sense of heat, fulness and throbbing, are felt for a few days, and there may be some little feverishness; but Dr. Houston has not seen or heard of any more serious effects from the remedy. The symptoms produced are usually so mild as not absolutely to require confinement to bed for more than a few hours; although, for many reasons, such confinement may often be desirable. On the third or fourth day, a purgative draught should be administered, when the bowels will be found to yield to the medicine, generally without either pain or prolapse of the rectum. The progress after this to healing is rapid, and free from any disagreeable symptoms.

A question may arise, as to whether, when two or more vascular tumors coexist, both or all should be touched at the same time. Such will generally be

* Dublin Journal, March.

the proper course for adoption. The severity of the remedy is moderate, and the great advantage of completing the operation at once, and of having the time for the accomplishment of the cure thereby materially abridged, will be secured by the adoption of this course. The circumstances of the case must, however, often determine such a matter more than any pre-concerted rules.

---

### Remarks on Outward Applications to Ulcers. By W. H. O. Sankey.*

On examining the composition of the more common medicinal applications to indolent ulcers, it would appear that, 1st, their energy on the animal tissue, and their caustic properties, are in direct ratio to the rapidity with which they part with their oxygen; and 2nd, the bases of those compounds which experience has taught us to prefer, are, when deprived of their oxygen, perfectly inert, and that their affinity for oxygen is comparatively slight.

Three qualifications, therefore, appear necessary to constitute a good outward application to an indolent ulcer. 1st. A substance containing a large proportion of oxygen, or of an electro-negative body. 2nd. A compound that will part with its oxygen to the animal organism moderately slowly. 3rd. A compound whose elements, when its oxygen is abstracted, possess no chemical or solvent powers on the tissues, nor poisonous effects on the body generally.

Guided by these views, Mr. Sankey is led to recommend the employment of various compounds to indolent ulcers, such as, the iodate of the protoxide of mercury, in the strength of ℈ss. to ʒss. to the ounce of lard; the iodide of starch; the oxide of silver; pectic acid applied as a poultice: the iodate of peroxide of mercury; the preparations of periodic acid, the bromates; succonates; the precipitate formed by tr. opii on Goulard water; mucic acid; alloxan; hydrated peroxide of iron, &c.

In angry and inflammatory ulcers, on the other hand, which are attended with increase of circulation, and, consequently, with excessive supply of oxygen, the oxygenated compounds would be prejudicial. Cold lotions or poultices, for chemical reasons, would be indicated; or, if ointments are used, those only containing a preponderance of electro-positive elements, as ung. cetacei, creosote, &c.; perhaps, also, the application of turpentine, or the non-oxygenated essential oils, as the ol. limon.

---

### Effect of Tartar Emetic on the Genital Organs.†

Mr. Griffiths relates the two following instances of the appearance of a copious crop of pustules all over the scrotum, after the application of the unguent. antimonii potass. tart. to other parts of the body, no eruption occurring in the situation where the remedy was applied.

In May, 1838, Mr. Griffith was consulted by a young man labouring under symptoms of incipient phthisis; an ounce of tartar emetic ointment was directed to be rubbed on the chest in the usual manner. In the course of a few days the whole of the scrotum was found to be covered with a dense crop of pustules far advanced towards suppuration; the patient was charged with having applied his

---

* Medical Gazette, March 10th.

† Prov. Med. Journn. Nov. 12th and Nov. 25th, 1842.

hands to the parts after using the ointment; this however he resolutely denied: the pain was soon relieved by cold saturnine lotions, anodyne fomentations, and emollient poultices, &c.

Soon after the above, the following case occurred:—

Mr. J. D. aged 39, a respectable farmer, consulted Mr. Griffith for some hepatic affection; he was, in addition to other treatment, directed to rub in, every four or six hours, a teaspoonful of an embrocation containing one drachm of tartar emetic; on the third day, a dense crop of pustules was found covering his scrotum; he also, like the other patient, protested that he had immediately washed his hands each time after using the ointment. In neither instance was there the slightest trace of any redness or irritation on the part to which the application was directed to be made. Mr. Griffith therefore concluded that "absorption had taken place, and some specific action exerted on or conveyed to the external covering of the scrotum, similar to what is observed in persons who have been laboring in mines containing a great proportion of arsenic, or those who have been exposed to the fumes arising from the consumption or volatalization of arsenious acid in various trades and manufactures."

Dr. Pereira, in his work on Materia Medica, remarks, "occasionally adventitious eruptions have appeared in other parts of the body, which have been ascribed to absorption of antimony into the system. But I believe, with Rayer, that they arise from the inadvertent application of the ointment to these parts." In these two cases, however, such does not appear to have been the fact.

Since the publication of the above, a letter has appeared from Mr. Pitt, of Mattishall, relating the following similar case.

J. D. was ordered to use the tartar emetic ointment, for chronic swelling of the knee-joint, after rheumatism, which soon produced a crop of painful pustules on the inner side of the knee, he was then directed to continue the application on the outer side; this soon produced a severe effect upon the scrotum, the pustules here assuming quite a confluent character. He denied having touched the part either with the ointment or his unwashed hands. The patient was of spare habit, and had his scrotum much relaxed by confinement to bed, which probably rendered it more susceptible of the action of the ointment; but whether it was carried to that organ by absorption, or was brought in immediate contact with it by means of the shirt, appears doubtful. Mr. Pitt has seen a similar affection on the scrotum of a lad fifteen years of age, whose mother had been rubbing croton oil over the abdomen, and had carefully avoided all other parts.

---

Excursions to some of the Principal Mineral Waters of England. By James Johnson, M.D. 1 Vol. Royal 12mo. pp. 160, price 5s. Highley, 1843.

As this work cannot be reviewed in this Journal, an extract or two, as specimens, are all that we can adduce.

### English and Foreign Spas.

"There are so many points of comparison and contrast between the foreign and domestic spas, that an ingenious casuist would have little difficulty in proving with much plausibility, the superiority of the German waters over the English, or the *latter* over the *former*, according to the party by which he was retained, or his own fancy. But the medical casuist should plead for truth, and not for victory—for the good of suffering humanity, not for self-interest. It must be confessed, in limine, that the English mineral waters cannot compete with the Continental, in strength, flavour, or high temperature. But it is not the most potent or the most palatable medicines that are the most useful or efficacious. It is the same with mineral waters. The Soolen-sprudel, at Kissengen, contains three times as much weight of ingredients as the Kochbrunnen, at Weisbaden, yet it is not one-tenth to useful as the latter. Many of the most celebrated waters of the Continent, as Pfeffers, Gastein, Teplitz, Wildbad, &c. have scarcely any active ingredients in their composition.

" But there is another important point to be taken into consideration. There is not one in one hundred of our British invalids requiring the remedial agency of a spa, who can afford either the time or expense necessary for a journey to even the nearest of the German mineral waters. The expense of an English passport, leaving aside the worry of getting it countersigned by the representatives of half the potentates of Europe, would pay the fare of a London citizen to the most distant spa in England ! It is very easy, but it is very useless, to tell a man with a large family and limited income, that venison and turtle are better fare than mutton and codfish—or, that Champagne and Burgundy are superior beverages to Marsalla and porter. Ordinary people must put up with ordinary provender—and many hundreds of our countrymen and women must be content with Bath instead of Baden—Harrogate in lieu of Aix-la-Chapelle—Cheltenham instead of Marienbad—and Tunbridge Wells as a substitute for Schwalbach or Bruckenau.

" For the real or supposed inferiority of British spas, the middle classes have some compensation, or at least consolation, in the saving of time and money—the security from a sea-voyage and sea-sickness—the trouble and expense of embarkations and debarkations—the scrambles at foreign hotels—the vexations of passports, police, and douanes—the rough roads, slow journies, and violent concussions—unaccustomed diet—ignorance of the language—the long and wide separation from friends and native home ; at a time, too, of sickness and anxiety. It must be acknowledged that these are only negative advantages—but HEALTH itself is no more than the absence of disease. These negative advantages of the home spa-goer, of middle rank, and which would be converted into positive evils abroad, are little felt by the aristocracy and the opulent classes of this country, who travel to the continental waters in their own carriages—with their own servants and couriers—and with every *portable* comfort which money can procure. To them the inconveniences alluded to, are, perhaps, more useful than otherwise, as breaking the monotony of their lives, dispersing ennui, and abstracting the mind of the invalid from his own gloomy meditations. To all these I would say, go and travel to the spas of Germany, and when you can swallow no more, ascend the Alps and take air instead of water—exercise instead of lounging—and occupy the mountain chalet rather than the splendid cursall.

" But has the home spa-goer no *positive* advantages over the continental pilgrim in pursuit of health ? I apprehend that he has—though he may not appreciate them unless he has crossed the Channel.

" Is the superb travelling, whether by rail, stage, or post, through a country, unequalled in Europe, for beauty, cultivation, and fertility—*nothing ?* Are Macadamized roads, on which we are neither blinded by dust, splashed with mud, or contused in body and limbs—*nothing ?* Is the comfortable hotel, where plain and wholesome food can be procured at ten minutes' notice, by day or by night—*nothing ?* Are beds, six feet in breadth, by eight in length—*nothing ?* Is the box-seat of a splendid stage-coach, which whirls us through the fresh air at the rate of 12 miles an hour, amid scenes and landscapes of exquisite beauty—*nothing ?*—There is, at all events, *nothing* of the kind on the Continent. Are the chalky cliffs, the boundless ocean, and the balmy atmosphere of our sea-girt shores, which may be approached in a few hours from any spa in

England—*nothing?* Is the daily post, through which we communicate with our family and friends—by means of which we can direct our concerns as easily and readily at Harrogate, as if we resided at Hampstead or Highgate—and all for one penny—*nothing?* He only who has experienced the tiring delays and heavy expences of foreign post-offices, can appreciate the blessings of the British penny post!

"Is the consciousness that, in the hour of peril, sickness, and distress, our nearest and dearest relations can fly to our succour in a few hours, even from the distant Metropolis—*nothing?*

"Finally, is there nothing consolatory in the anticipation that, should the hand of death press upon us, when absent from our family in quest of health, we shall have the consolations of religion administered by our own clergy, and our native soil as a lasting sepulchre where our ashes may repose with those of our friends and relations?" 3.

### Hydropathy.

"But it is in chronic diseases, that is, diseases without any evident fever or inflammation, acute or subacute, that hydropathy flourishes, and its disciples exult; and yet it is in this very class of afflictions, large and multifarious as it is, that the 'water-cure,' as it is erroneously called, produces what its name expresses—the 'Water-disease'—and slays its thousands—not, indeed, in an open, obvious, and sudden manner, but in a slow, insidious, and masked character, when the victim is totally unconscious of the precipice to which he is advancing, and over which he will inevitably be hurled. Those who have seen most of human maladies, are well aware that the *causes* of chronic diseases, as the very word (chronic, from *chronos*, time) imports, are slow and gradual in implanting themselves in the constitution, and, when once fixed, are equally, if not more slow in their removal. In fact, it is known to every practitioner of experience that not one chronic disease in ten can be cured at all, and that the most we can expect is a mitigation of suffering. But there are certain classes of maladies—for instance, gout, rheumatism, rheumatic gout, tic douloureux, &c., which, though thoroughly constitutional, and whose *causes* have been years accumulating, are yet of a migratory nature, suddenly shifting their seat from a vital to an unimportant organ, and, *vice versa*, from a foot or a wrist to the stomach or heart. Now, it is an undoubted fact, that when a translation or metastasis of a chronic or sub-acute affection, as of gout or rheumatism, suddenly takes place from the exterior of the body, whether spontaneously or by the force of medicine, the malady takes up its seat in some internal organ;—but as internal organs, as the heart, liver, brain, &c. are not naturally sensitive of pain, the metastasis is very often taken for a cure, and the malady preys for a long time on a vital part, without suspicion, till it reaches a certain height, when the disease not only reveals itself, unequivocally, by pain and suffering, but is now totally beyond the power of art!! Nature will not be cozened by the ingenuity of man. For a long time she counteracts the deleterious effects of morbific causes, whether applied by ourselves, or unavoidably occurring, and guards vital organs by throwing the onus on external parts, as is familiarly exemplified in gout. But, when we thwart these salutary efforts of Dame Nature, by violently repelling the pain, inflammation, stiffness, or swelling from the hands and feet, by cold applications, heroic doses of colchicum, &c. then we lay the foundation, directly or remotely, for serious or even fatal maladies of some of the internal viscera! In this drama Hydropathy is now playing an important part, and we are now in almost daily habits of seeing the precious fruits of the 'water-cure,' in the shapes of furuncles, carbuncles, dropsy, and hypertrophy of the heart—internal abscesses, and other grave consequences. These, however, will not be heeded—at least they will not make much impression on the public, till some great personage dies suddenly under the Hydropathic process, when the bubble will burst, and the 'WATER-CURE' shrink within its natural and *salutary* boundaries. I say *salutary;* because, in a large class of patients, labouring under dyspeptic, hypochondriacal, and nervous complaints, the early hours, the abstinence from wine and other stimulants, the vigorous exercises and the external and internal use of water itself, may prove more efficacious than medicine, indolence, and repletion at home. But let those who are subject to rheumatism, gout, erysipelas, tic douloureux, or any migratory disease, beware of the wet sheets, the plunge into cold water while perspiring, and the enormous ingurgitation of cold water, if they wish to avoid enlargement of the heart, chronic inflammation of the lungs, congestion of the liver, dropsy of chest, or the affliction of JOB!" 160.

---

CASE OF SECTION OF TENDO-ACHILLIS, OF ADDUCTOR LONGUS, OF SARTORIUS AND RECTUS FEMORIS, AND OF TENDON OF PSOAS AND ILIAC MUSCLES, IN THE SAME SUBJECT. By JOSEPH SARGENT, M.D., of Worcester.*

The division of muscles and tendons is on the decline in this country. In Boz's Essays, a racy account is given of the epidemic passion for gas-lights and plate-glass, that affected successively the gin-shopkeepers, chemists, linen-drapers, and house-decorators of the metropolis. The rage amongst the surgeons for dividing tendons has been just as frantic and unaccountable. Sober people looked on and wondered what could be the matter, whilst gentlemen were running about town, brandishing tenotomes of all shapes and cuts, and poking them into persons' eyes, heels, necks, and buttocks, with equal determination and impartiality. The influenza raged violently for a time, but its virulence has sensibly abated in London, and it has lapsed into a harmless and chronic state.

Over the water, however, it is otherwise. Our American friends have suffered from the disease in its aggravated form, and the symptoms have been peculiarly distressing. Nor are they yet altogether subdued. Restlessness and pervigilium, an irresistible impulse to tenotomise, and a hallucination with regard to the benefits obtained by the unfortunate patients, are the prominent symptoms of a malady which at the very least is still subacute.

In a case before us Dr. Sargent gives the following account of his exploits.

April 27th.—"I divided the tendo-Achillis by the common subcutaneous operation, cutting from above downwards. The twitching of muscles of calf, to which patient had been so long accustomed, continued for several hours after the operation, and was painful. It then ceased, and patient became unusually quiet."

May 3d.—"Patient doing well, I divided the adductor longus, at a distance of about an inch and a half from the pubis—entering knife from inside of femur and cutting downwards. A small artery was divided, from which there was some hæmorrhage at the time of the operation. Patient continued well through first, second and third days after operation; and on the fourth was bound to a mattrass by a strap passing over his hips, confining pelvis, and one over each thigh, so as to keep these in extension and abduction as much as possible."

May 19th.—"Thigh being held in abduction and extension, I divided the sartorius and rectus femoris muscles, passing a tenotome, slightly convex on its edge, under the skin just behind and below the anterior superior spinous process of ilium, and sliding it about parallel with Poupart's ligament (so as to keep

---

* The New England Quarterly Journal of Medicine and Surgery, No. 1. July, 1842.

the probable parallel of recurrent arteries) till it had passed inner border of rectus, and this was divided by passing in the knife a second time. The divided ends of the muscles parted to distance of two or three inches. There was some bleeding, and I did up the hip in compresses and a snug bandage."

This would not do, and Dr. Sargent felt that nothing short of the tendon of the psoas and iliacus would suit him. The femoral artery might be divided, it is true, but what of that?

"The operation could leave the patient in no worse state than his present; and if successful, promised great results. Even if the femoral artery should be cut and have to be tied, the muscular tension might be removed at the same time, and the operation, at the worst, would be of a class with those performed even in our day, and by Dupuytren in torticollis, a deformity of infinitely less importance."

As the epigram on Dr. Lettsom says—

> " If after that they choose to die,
> What's that to me, I let's em.

June 19th.—" In presence of Dr. Green, Dr. Heywood, and Mr. B. F. Heywood, I passed in a pointed tenotome from outside of thigh, about an inch and a half below anterior superior spinous process of ilium, and slid it under the skin, in a direction parallel with Poupart's ligament, for the space of about three inches. Withdrew this and followed with probe-pointed tenotome; but I was in the field of last operation, and could not get through the close knit cellular tissue far enough. I had endeavoured to operate here so as to avoid the profunda and the recurrent arteries. Withdrew the knife, and commenced in same way about an inch and half below, and a little in front of last aperture. I kept my left thumb nail hard upon the edge of the femoral artery, a position which I had obtained for it while the thigh was in a state of flexion, and passed the knives in succession quite up to this nail. As the probe-pointed one reached it, I turned it so as to cut downwards, and Dr. Green made efforts in the way of extension. I trusted to the curve described by the blade as I turned it on the axis at the junction of the blade with the handle, for a progressive removal of the knife from the femoral artery. The section required considerable force, but the tension yielded after several cracks, at each of which Dr. Green said that he felt the limb give considerably, except at the last, which brought the blade hard on to the bone, and was followed, on withdrawal of the knife, with a very large jet of blood, such as to quite bespatter the operator from head to foot. Thick compresses were bound down over the incision, and a roller laid over the femoral artery and in its course, and confined by a firm bandage. Patient said that whole limb felt dead quite down to foot. A few hours after, when he had got over the fright from the operation, he said, 'how queerly it felt in my back when you cut the cord; it was all loose there, and now it goes right back.' His thigh too, he said, felt all free."

Of course there was trouble, hæmorrhage, extension, pain and all that sort of thing, but, bye and bye, the patient was put in "harness" and he got well enough to go home, and, as the operator *heard*, to "improve a good deal."

And so ends this eventful history, in which the patient was brought by the aid of art just to death's door, but not actually pushed through it.

